# Anti-glomerular Basement Membrane Glomerulonephritis: A Study in Real Life

**DOI:** 10.3389/fmed.2022.889185

**Published:** 2022-07-05

**Authors:** Marina Sánchez-Agesta, Cristina Rabasco, María J. Soler, Amir Shabaka, Elisabeth Canllavi, Saulo J. Fernández, Juan M. Cazorla, Esperanza López-Rubio, Ana Romera, Sergio Barroso, Ana Huerta, Leonardo Calle, Milagros Sierra, Patricia Domínguez-Torres, Manuela Moreno-Ramírez, Sara Afonso, Victoria Mascarós, Armando Coca, Mario Espinosa, Spanish Group for the Study of Glomerular Diseases (GLOSEN)

**Affiliations:** ^1^Department of Nephrology, Hospital Universitario Reina Sofía, Córdoba, Spain; ^2^Department of Nephrology, Hospital Vall d’Hebron, Barcelona, Spain; ^3^Department of Nephrology, Hospital Clínico San Carlos, Madrid, Spain; ^4^Department of Nephrology, Hospital 12 de Octubre, Madrid, Spain; ^5^Department of Nephrology, Hospital Universitario Insular de Gran Canaria, Las Palmas, Spain; ^6^Department of Nephrology, Hospital Universitario Puerta del Mar, Cádiz, Spain; ^7^Department of Nephrology, Hospital General de Albacete, Albacete, Spain; ^8^Department of Nephrology, Hospital General Universitario de Ciudad Real, Ciudad Real, Spain; ^9^Department of Nephrology, Hospital Universitario de Badajoz, Badajoz, Spain; ^10^Department of Nephrology, Hospital Universitario Puerta de Hierro Majadahonda, Madrid, Spain; ^11^RedinRen ISCIII RETYC 16/009, Hospital Universitario Puerta de Hierro Majadahonda, Madrid, Spain; ^12^Department of Nephrology, Complejo Asistencial de Segovia, Segovia, Spain; ^13^Department of Nephrology, Hospital San Pedro, Logroño, Spain; ^14^Department of Nephrology, Hospital Universitario Fundación Alcorcón, Madrid, Spain; ^15^Department of Nephrology, Hospital Juan Ramón Jimenez, Huelva, Spain; ^16^Department of Nephrology, Hospital Universitario La Paz, Madrid, Spain; ^17^Department of Nephrology, Hospital Francesc Borja de Gandía, Valencia, Spain; ^18^Department of Nephrology, Hospital Clínico Universitario de Valladolid, Valladolid, Spain

**Keywords:** anti-glomerular basement membrane disease, crescents, glomerulonephritis (GN), end-stage kidney disease (ESKD), kidney survival, plasma exchange

## Abstract

**Introduction:**

Anti-glomerular basement membrane (anti-GBM) disease is a severe entity with few therapeutic options including plasma exchange and immunosuppressive agents. The aim of this study was to analyze the clinical and pathological features that predict the evolution of end-stage kidney disease (ESKD) and the kidney survival in a cohort of patients with anti-GBM disease with renal involvement in real life.

**Methods:**

A retrospective multicentre observational study including 72 patients from 18 nephrology departments with biopsy-proven anti-GBM disease from 1999 to 2019 was performed. Progression to ESKD in relation to clinical and histological variables was evaluated.

**Results:**

Creatinine at admission was 8.6 (± 4) mg/dL and 61 patients (84.7%) required dialysis. Sixty-five patients (90.3%) underwent plasma exchange. Twenty-two patients (30.6%) presented pulmonary hemorrhage. Kidney survival was worse in patients with creatinine levels > 4.7 mg/dL (3 vs. 44% *p* < 0.01) and in patients with > 50% crescents (6 vs. 49%; *p* = 0.03). Dialysis dependence at admission and creatinine levels > 4.7 mg/dL remained independent significant predictors of ESKD in the multivariable analysis [HR (hazard ratio) 3.13 (1.25–7.84); HR 3 (1.01–9.14); *p* < 0.01]. The discrimination value for a creatinine level > 4.7 mg/dL and 50.5% crescents had an area under the curve (AUC) of 0.9 (95% CI 0.82–0.97; *p* < 0.001) and 0.77 (95% CI 0.56–0.98; *p* = 0.008), respectively. Kidney survival at 1 and 2 years was 13.5 and 11%, respectively. Patient survival at 5 years was 81%.

**Conclusion:**

In real life, patients with severe anti-GBM disease (creatinine > 4.7 mg/dL and > 50% crescents) remained with devastating renal prognosis despite plasma exchange and immunosuppressive treatment. New therapies for the treatment of this rare renal disease are urgently needed.

## Introduction

Anti-glomerular basement membrane (anti-GBM) disease is a rare and severe glomerular condition. The disease is characterized by the presence of autoantibodies against the non-collagenous domain of the alpha 3 chain of type IV collagen [α3(IV)NC1] in the glomerular and alveolar basement membranes (1). The incidence is estimated to be 0.5–1.5 cases per million per year (2). This entity usually leads to rapidly progressive glomerulonephritis and has an incidence of alveolar hemorrhage that varies between 23 and 40% (3, 4). In most cases, immunofluorescence shows linear immunoglobulin G (IgG) deposits along the glomerular basement membrane (GBM) and serum anti-GBM autoantibodies can be detected (5, 6). The co-existence of antineutrophil cytoplasmic antibodies (ANCA) with anti-GBM antibodies has been detected in 21–38% of patients (7–10). Outcomes in double and single positive patients have conflicting findings (9, 11). Around 80–100% of patients who require dialysis at admission develop end-stage kidney disease (ESKD) (4, 12–14). Oligoanuria, the high percentage of crescents in renal biopsy, and dialysis dependence at admission have been associated with poor prognosis in this disease (6, 9, 12, 13). The recurrence rate in transplants is approximately 50% when transplantation is performed while circulating antibodies are still present.

Plasma exchange and immunosuppressive agents, particularly cyclophosphamide and corticosteroids, are the main therapy for treating this disease (15). Current treatment protocols are similar to the one initially described in 1976 (16). The current Kidney Disease Improving Global Outcomes (KDIGO) guidelines recommend initiating immunosuppression with cyclophosphamide and corticosteroids plus plasmapheresis in all patients with anti-GBM glomerulonephritis except those who are dialysis-dependent at presentation, have 100% crescents in an adequate biopsy sample and do not present pulmonary hemorrhage (15). These recommendations are based on a single randomized prospective study and several retrospective series (12, 17). Therefore, more studies are needed to further improve patient treatment. In addition, it is uncertain if these recommendations are implemented in daily clinical practice. The aim of this study was to analyze the clinical and pathological features that predict the evolution of ESKD and kidney survival in a cohort of anti-GBM patients with renal involvement in real life. Kidney outcomes in double positive patients (anti-GBM and ANCA antibodies) and kidney evolution after receiving a kidney transplant were also evaluated.

## Materials and Methods

This is a retrospective, multicentre, observational study including 72 patients from 18 nephrology departments belonging to the Spanish Group for the Study of Glomerular Diseases (GLOSEN). Data from patients with anti-GBM disease from 1999 to 2019 were collected. All included patients had a renal biopsy performed. Anti-GBM disease was defined as biopsy-proven crescentic glomerulonephritis and positive glomerular linear IgG staining detected by immunofluorescence or the presence of serum anti-GBM antibodies in serum. Anti-GBM and ANCA serology was tested in all the patients. Patients who met the diagnosis of anti-GBM disease and also presented a positive ANCA serology were included in a double positive cohort.

Biopsy specimens were analyzed by an expert pathologist at each center using optical microscopy and immunofluorescence. Six specimens for immunofluorescence did not contain assessable glomeruli. The percentage of crescents was evaluated by light microscopy. The fragment used for immunofluorescence was processed with immunoreactants for IgG, IgA, IgM, complement (C3, C1q), fibrinogen, and light chains (kappa/lambda). Visualization was performed using a dark-field microscope with ultraviolet light. Patients were classified according to their percentage of crescents.

Demographic, clinical, and analytical variables were evaluated at the time of kidney biopsy. In each patient, the reference point was established at the time of biopsy. Follow-up time was established as the interval between the biopsy and the last visit, kidney replacement therapy, or death. Development of kidney failure was evaluated. The clinical course of patients who received a renal transplant was also assessed.

Standard therapy consisted in plasma exchange, oral or pulses of steroids (approximately 1 mg/kg of body weight per day) and oral cyclophosphamide (2–3 mg/kg per day) or intravenous cyclophosphamide (15 mg/kg every 2 weeks for 2–3 months). Plasma exchange (2–4 L) was performed either daily or every other day for at least six sessions or until anti-GBM antibody was undetectable. The plasma volume was calculated following the formula: “Estimated plasma volume (in liters) = 0.07 × weight (kg) × (1 - hematocrit)”(18). Human albumin (5%) was used as replacement material. Fresh frozen plasma was used in patients with alveolar hemorrhage or after recent renal biopsy.

### Statistical Analysis

Categorical variables were expressed as proportions (%) and differences were assessed using the chi-squared test. Continuous variables were expressed as means ± standard deviation (SD) or medians (interquartile range) and were compared using the Student’s *t*-test. A cox regression model was used to estimate predictors of kidney survival. Kidney survival was analyzed using the Kaplan–Meier method and log-rank test.

Receiver operating characteristic (ROC) curves were plotted and the area under the curve (AUC) was estimated to assess the predictive performance of serum creatinine and crescents in discriminating ESKD. In addition, the best discrimination limit for these levels was explored by the maximum of Younden’s index (sensitivity + specificity – 1). This corresponds to the value where sensitivity plus specificity is maximized. All computations were carried out using SPSS 25.0 for Windows (SPSS Inc., Chicago, IL, United States). A *p*-value less than 0.05 was considered statistically significant.

## Results

### Baseline Characteristics

Our study included 72 patients from 18 Spanish centers. The demographic and analytical characteristics of the patients are shown in Table 1.

**TABLE 1:** Demographic and analytical characteristics of the patients.

	Total (*n* = 72)	Anti-GBM (*n* = 41)	Double positive (*n* = 31)	*P*-value
Female, *n* (%)	40 (55.6)	21 (52.5)	19 (61.0)	0.48
Age (years)	58.7 (± 19)	52.0 (± 21)	67.7 (± 12)	0.01
Creatinine at admission (mg/dl)	8.6 (± 4.0)	7.4 (± 3.5)	10.4 (± 5.0)	0.01
eGFR (CKD-EPI) at admission (ml/min)	10.4 (± 14.8)	13.7 (± 18.5)	6.0 (± 5.7)	0.03
Hemoptysis, *n* (%)	22 (30.5)	12 (29.0)	10 (32.0)	0.80
AntiGBM antibodies, *n* (%)	67 (93)	36 (88)	31 (100)	0.50
Crescents, *n* (%)	80.0 (± 25.0)	78.0 (± 25.7)	89.5 (± 25.2)	0.52
IgG lineal deposit, *n* (%)	66 (92.0)	37 (95.0)	29 (93.5)	0.90
Corticosteroids, *n* (%)	71 (98.6)	41 (100)	30 (96.8)	0.42
Cyclophosphamide, *n* (%)	65 (90.3)	38 (92.7)	27 (87.1)	0.45
Plasma exchange, *n* (%)	65 (90.3)	37 (90.2)	28 (90.3)	0.90
Number of plasma exchange	10 (3–29)	10 (3–29)	8 (3–19)	0.35
Dialysis at presentation, *n* (%)	61 (84.7)	32 (78.0)	29 (93.5)	0.10
ESKD, *n* (%)	63 (87.5)	36 (87.8)	27 (87.0)	0.91
Creatinine at follow-up (mg/dl)	2.1 (± 0.82)	2.2 (± 1.01)	1.9 (± 0.43)	0.52
Transplantation, *n* (%)	18 (25.0)	12 (27.5)	6 (19.4)	0.57
Creatinine at follow-up in transplantation (mg/dl)	1.26 (0.8–4.6)	1.2 (0.9–4.6)	1.85 (0.8–3.2)	0.52
Deaths, n (%)	17 (22.2)	9 (22.0)	7 (22.6)	0.91

*AntiGBM, anti-glomerular basement; ESKD, end-stage kidney disease.*

The mean percentage of crescents in each renal sample was 75.6% (± 25). Twenty-six patients (36%) had 100% crescents in renal biopsy. As expected, the mean serum creatinine level was higher in the group of patients with ≥ 50% crescents than the group with < 50% crescents (9.16 mg/dL vs. 5.43 mg/dL; *p* = 0.03) (Supplementary Figure 1). In 66 patients (92%), immunofluorescence staining for IgG in the kidney biopsy sample showed linear deposits along the GBM.

Anti-GBM antibodies were positive in 67 patients (93%). Thirty-one of these patients (44.4%) were also positive for ANCA (“double positive”); 28 (90.7%) were positive for P-ANCA and 3 (9.3%) were positive for C-ANCA. Double positive patients were older than single positive patients (67 vs. 52 years, *p* = 0.01). In double positive patients, mean creatinine level was higher at admission (10.35 vs. 7.38 mg/dL; *p* = 0.01). However, no differences were observed in dialysis dependence at admission and ESKD development.

**FIGURE 1:**
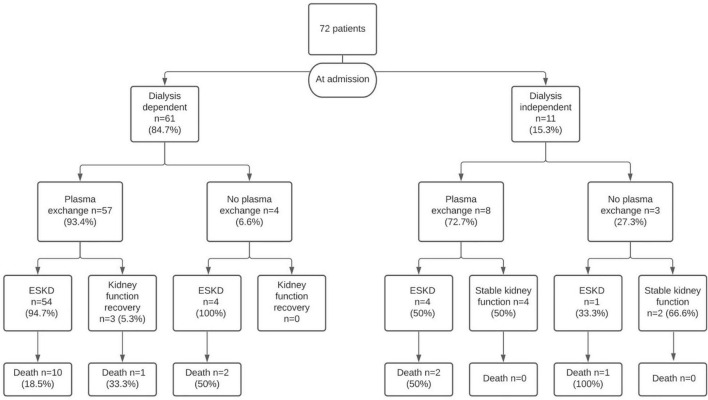
The flowchart shows the patient’s outcomes according to the treatment received.

Twenty-two patients (30.6%) presented pulmonary hemorrhage. Patients with pulmonary hemorrhage were younger than those without pulmonary hemorrhage (48 vs. 63 years; *p* = 0.01). These patients received more plasma exchange sessions (12 vs. 9; *p* = 0.03). No differences were observed in serum creatinine at admission, crescents, dialysis at presentation, evolution to ESKD, or death between patients with and without pulmonary hemorrhage (Table 2). Sixty-one patients (84.7%) required dialysis at admission (Figure 1). Three of these 61 patients recovered kidney function during follow-up. The median days of dialysis in these 3 patients since their first dialysis was 33 (24–33) days. Antibodies titer of these patients were 1/640 and 181 UI/mL for P-ANCA and 6.73; 2.9 and 148 UI/mL for anti-GBM antibodies, respectively. Eleven patients (15.3%) did not require dialysis at admission. The median follow-up of dialysis-independent patients was 31 (10.5–86.8) months. Five of these patients developed ESKD during follow-up.

**TABLE 2:** Clinical and pathologic characteristics of patients with and without pulmonary hemorrhage.

	Pulmonary hemorrhage (*n* = 22)	Not pulmonary hemorrhage (*n* = 50)	*P*-value
Female, *n* (%)	12 (54.4)	30 (60.0)	0.79
Age (years)	48 (± 27)	63 (± 17)	0.01
Creatinine at admission (mg/dl)	8.6 (± 4.2)	8.5 (± 5.0)	0.90
AntiGBM antibodies, *n* (%)	20 (91)	47 (94)	0.63
ANCA, *n* (%)	11 (50)	21 (42)	0.61
Smoking (%)	9 (40.9)	15 (30)	0.26
Crescents, *n* (%)	72 (± 31)	77.2 (± 23)	0.40
IgG lineal deposit, *n* (%)	21 (95.5)	45 (93.8)	0.90
Plasma exchange, *n* (%)	21 (95.5)	44 (88.0)	0.42
Number of plasma exchange	12 (± 5.0)	9 (± 3.8)	0.03
Dialysis at presentation, *n* (%)	18 (81.8)	43 (86.0)	0.72
ESKD, *n* (%)	19 (86.4)	44 (88.0)	0.91
Deaths, *n* (%)	5 (22.7)	11 (22.0)	0.58

*ANCA, antineutrophil cytoplasmic antibodies; AntiGBM, anti-glomerular basement; ESKD, end-stage kidney disease.*

Lastly, a total of 63 patients (87.5%) developed ESKD, in most cases present at disease onset. Nine patients (12.5%) remained dialysis free with a mean serum creatinine level of 2.09 (± 0.82) mg/dL. Two patients had renal survival without plasma exchange. Antibodies titer in these two patients were 80.5 UI/mL and 52 UI/mL for anti-GBM and negative for ANCA. Seventeen patients (22.9%) died during follow-up. Eighteen patients (25%) underwent a renal transplant. The median follow-up after transplants was 78.3 (19.3–169.1) months. None of them exhibited evidence of graft relapse.

### Treatment

Sixty-five patients (90.3%) underwent plasma exchange and the majority received immunosuppression treatment which in most cases included steroids and cyclophosphamide; 2 cases (2.77%) were treated with rituximab.

Only 7 of the patients (10.8%) who underwent plasma exchange did not develop ESKD (Table 3). The median serum creatinine at the admission of these patients was 3.9 (2.6–4.6) mg/dL, and the median of crescents was 50% (25–100). Patients number 1 and 3 (Table 3) had an unexpected evolution. These patients (6 and 1.8 mg/dL creatinine at admission and 75 and 100% cellular crescents, respectively) were treated with plasma exchange, required dialysis for 30 days, and recovered renal function during follow-up.

**TABLE 3:** Patients who undergo plasma exchange and not developed ESKD.

Patient	Creatinine at admission (mg/dl)	Dialysis at presentation	Crescents, n (%)	Creatinine at follow up (mg/dl)
1	6.0	Yes	75	1.8
2	4.6	No	25	2.0
3	1.8	Yes	100	2.4
4	2.6	No	40	1.4
5	3.9	Yes	50	1.9
6	2.7	No	50	1.4
7	4.0	No	47	3.6

Seven patients (9.7%) did not undergo plasma exchange. Of these patients, five (71.4%) developed ESKD. The 2 patients who did not progress to ESKD presented, respectively, 14 and 30% crescents in renal biopsy and creatinine levels at admission of 1.0 and 2.8 mg/dL.

In summary, the 9 patients who did not develop ESKD during follow-up had a median serum creatinine level of 2.8 mg/dL at admission and a median of 47% crescents.

### Predictors of End-Stage Kidney Disease

**FIGURE 2:**
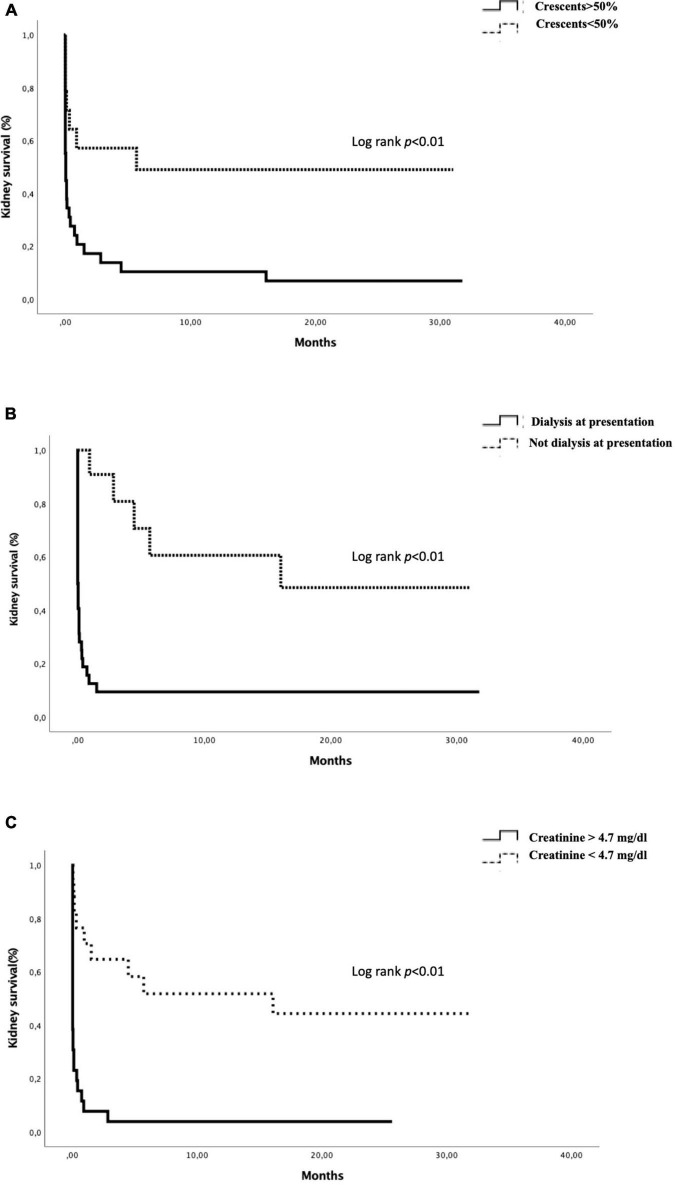
Actuarial renal survival by Kaplan–Meier curve according to the presence of > 50% of crescents **(A)**, dialysis at presentation **(B)**, and creatinine > 4.7 mg/dl **(C)**.

In the Kaplan-Meier analysis, kidney survival at 1 and 2 years was 13.5 and 11%, respectively. Kidney survival at 2 years was worse in patients with > 50% crescents on renal biopsy than in patients with < 50% of crescents in kidney biopsy (6 vs. 49%; *p* = 0.03) (Figure 2A). Kidney survival differed significantly between patients who required dialysis at presentation and those that did not (9 vs. 48%; *p* < 0.01) (Figure 2B). Kidney survival was also worse in patients with creatinine > 4.7 mg/dL at admission (3 vs. 44%; *p* < 0.01) (Figure 2C). Double positive patients had a similar kidney survival than single positive patients at 1 year (23 vs. 21; *p* = 0.4).

A Cox regression model was developed (Table 4). Creatinine at admission, creatinine > 4.7 mg/dL at admission, > 50% crescents in the renal biopsy sample, and dialysis at presentation were associated with ESKD in the univariable Cox regression. Dialysis dependence at admission and creatinine > 4.7 mg/dL at admission remained independent significant predictors of ESKD in the multivariable analysis [hazards ratio (HR) 3.13 (1.25–7.84); HR 3 (1.01–9.14); *p* < 0.01].

**TABLE 4:** Prognostic parameters of ESKD.

	Univariable analysis		Multivariable analysis	
	HR (95% CI)	*P*-value	HR (95% CI)	*P*-value
Age	1.004 (0.99–1.02)	0.60	1.002 (0.98–1.02)	0.85
Hemoptysis	1.02 (0.59–1.78)	0.91	1.01 (0.54–1.89)	0.96
Creatinine at admission	1.05 (1.01–1.11)	0.02	1.03 (0.96–1.10)	0.42
Crescents	1.01 (0.99–1.02)	0.09	1.003 (0.97–1.02)	0.84
Crescents > 75%	1.15 (0.86–2.51)	0.15	1.03 (0.29–3.59)	0.95
Crescents > 50%	1.98 (1.01–3.89)	0.04	1.39 (0.68–2.83)	0.36
Creatinine at admission > 4.7 mg/dl	3.62 (1.62–8.07)	<0.01	2.37 (1.01–5.60)	0.049
Plasma exchange	1.21 (0.43–3.62)	0.70	0.72 (0.24–2.14)	0.56
Double positive	1.02 (0.61–1.70)	0.91	0.71 (0.38–1.29)	0.26
Dialysis at presentation	4.91 (1.67–14.45)	<0.01	3.15 (1.17–10.37)	0.04

**FIGURE 3:**
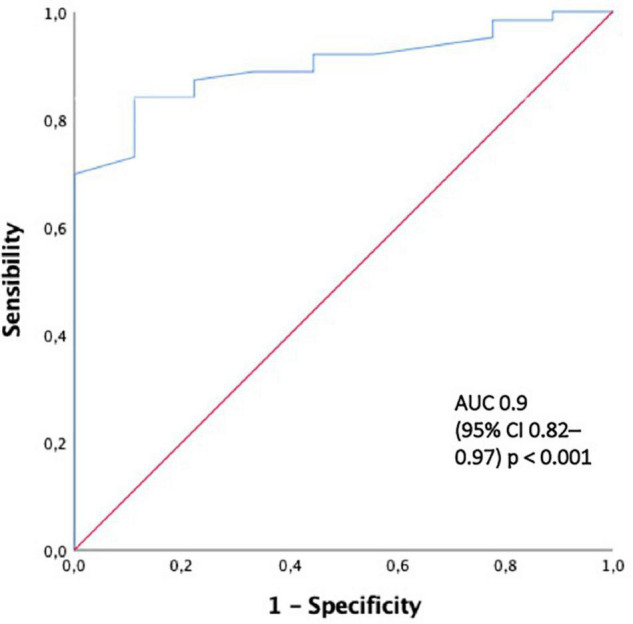
The ROC curve for predicting end-renal stage disease development with creatinine al presentation > 4.7 mg/dl. AUC area under the curve, ROC curve receiver-operating characteristic curve.

**FIGURE 4:**
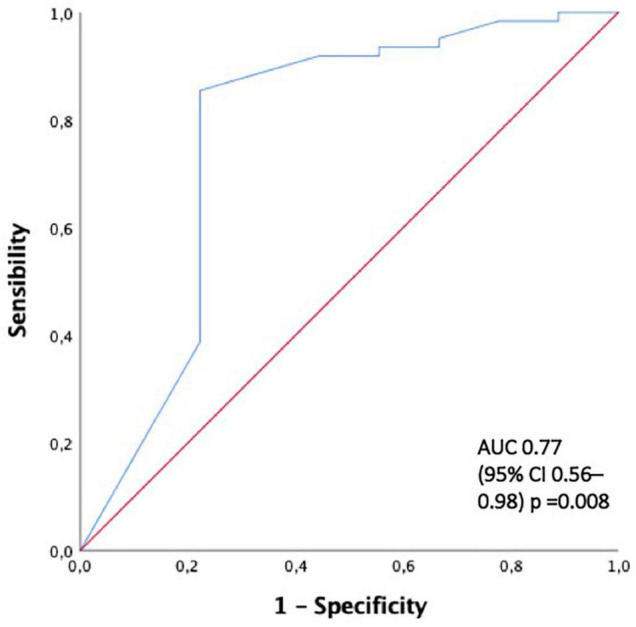
The ROC curve for predicting end-renal stage disease development with > 50% of crescents. AUC area under the curve, ROC curve receiver-operating characteristic curve.

The ROC curves for a serum creatinine level of 4.7 mg/dL and 50.5% crescents were 0.9 and 0.77, respectively (Figures 3, 4).

Patient survival at 1 and 5 years was 88 and 81%, respectively. There was no difference in patient survival between patients with double positivity, lung hemorrhage development, plasma exchange treatment, and ESKD development.

## Discussion

Anti-GBM disease is a poor prognostic entity in terms of renal and patient survival. To our knowledge this is the first multicentre observational study in real life of patients with anti-GBM glomerulonephritis in our country. KDIGO guidelines do not recommend immunosuppression and plasma exchange in patients who require dialysis at admission and have 100% crescents on renal biopsy without pulmonary hemorrhage (15). However, in real life, this recommendation is difficult to follow. In our study, most of the patients started treatment before renal biopsy results were available and a low percentage depicted 100% crescents on renal biopsy (36%). For that reason, most patients with anti-GBM glomerulonephritis are currently treated with plasma exchange and its impact on long-term renal survival is unknown. In the present study, plasma exchange was used in 90.4% of cases and the first-year kidney survival was only 13%. The kidney survival rate in our study is worse than previously reported. At 1-year follow-up, reported kidney survival rates range from 16 to 53% (3, 4, 12, 13, 19–21). Previous studies have shown creatinine at admission, oliguria or anuria, dialysis dependence at admission, and percentage of crescents on kidney biopsy to be predictors of worse kidney survival (4, 14, 19). In the present study, a creatinine level > 4.7 mg/dL at admission, > 50% crescents in renal biopsy sample, and dialysis at presentation were associated with ESKD. Likewise, the ROC curve shows that a creatinine level > 4.7 mg/dL at presentation is the major discriminant factor for the development of ESKD. According to our study, a serum creatinine level > 4.7 mg/dL can be considered an independent predictor of kidney failure despite the use of plasma exchange in this disease. Intensive therapy with immunosuppression and plasma exchange in this entity remains controversial. Levy et al. reviewed 71 patients treated with plasma exchange and an immunosuppressive regimen, patients with a creatinine concentration less than 5.65 mg/dL had 100% patient survival and 95% kidney survival rate at 1 year. In patients with severe renal failure requiring immediate dialysis, these values were 65 and 8%, respectively (12). In the retrospective review of 43 patients by Alchi et al., kidney survival was 16% at 1-year follow-up and the authors found similar patient and kidney survival in those who received intensive therapy and those who had minimal or no treatment (19). Recently, van Daalen et al. found that patients who were dialysis-dependent at presentation and had 100% cellular crescents at biopsy did not recover kidney function. They suggest that patients who are dialysis-dependent at admission and patients with either > 50% globally sclerotic glomeruli or 100% crescents do not benefit from intensive treatment and must receive a conservative regimen (4). On the basis of these studies, the KDIGO guidelines currently recommend that dialysis-dependent patients with 100% cellular crescents in an adequate biopsy sample who do not present lung hemorrhage can be exempt from intensive treatment with plasma exchange, corticosteroids, and cyclophosphamide (15). However, these recommendations have a questionable presence in daily clinical practice, in most cases, immunosuppressive treatment and plasma exchange are used, regardless of these factors. One surmise that this may be in part related to the kidney biopsy diagnosis delay.

In our study, patients (*n* = 9) who did not develop ESKD had a lower percentage of crescents (47%) and lower serum creatinine at admission (2.8 mg/dL), regardless of whether they received plasma exchange treatment. Therefore, we think that renal prognosis is mainly associated with kidney function at presentation, dialysis dependence at admission, and percentage of crescents on the kidney biopsy sample. Immunosuppressive treatment and plasma exchange are the treatments indicated in these patients. In concordance with previous authors, we found that worse renal function, dialysis dependency, and a low percentage of normal glomeruli were associated with poor kidney outcomes in anti-GBM GN independently of the treatment. Altogether these results suggest that in patients with severe renal anti-GBM plasma exchange does not offer a benefit in terms of ameliorating renal survival; however, in our study its effect on patient survival is unknown. New therapies for the treatment of this rare renal disease are urgently needed. An Open-Label Phase 2 Study to Evaluate the Efficacy and Safety of the endopeptidase IdeS (Inmunoglobulin G degrading enzyme of Streptococcus pyogenes) (Imliflidase) added to plasma exchange in non-dialysis dependent patients with anti-GBM disease has been performed and demonstrated that Imlifidase treatment was associated with an overall renal survival of 67% at 6 months (22).

Forty-four percent of our patients were double positive. The coexistence of ANCAs with anti-GBM antibodies is common. In some series, almost half of patients with anti-GBM disease have detectable ANCA [usually myeloperoxidase (MPO)] (23). Surprisingly, we were not able to demonstrate a worse renal outcome in patients with double positivity antibodies as compared to the single positivity ones. Previous studies have also compared outcomes in double and single positive patients with conflicting findings. Some studies have reported better outcomes in double positive patients (3, 9, 11, 24), while others have reported similar or worse outcomes in these patients (13, 19, 25–28). In our series, double positive patients had higher creatinine levels at admission than single positive patients. However, both groups had similar dialysis dependence at presentation and ESKD development. The presence of pulmonary hemorrhage was also comparable in both groups.

The incidence of recurrent linear IgG staining in transplantation is rare. The Australia and New Zealand Dialysis and Transplant Registry (ANZDATA) study found that 6 out of 449 (2.7%) patients developed biopsy-proven recurrent anti-GBM disease, with graft failure in 2 cases (3, 29). In the present study, patients with anti-GBM disease who underwent a renal transplant did not exhibit evidence of graft relapse and kidney survival was excellent, in line with previous results (29, 30).

## Conclusion

In conclusion, creatinine levels higher than 4.7 mg/dL, dialysis dependence at admission, and the presence of > 50% crescents on renal biopsy are associated with a worse renal outcome. In this study, kidney survival did not improve with plasma exchange, but the impact of this strategy on patient survival is unknown. Collaborative and multicentric randomized clinical trials are urgently needed for delineating the future treatment of this rare and poor prognostic glomerular disease. There is no evidence of clinical relapse in renal transplant patients with anti-GBM disease and long-term renal survival was excellent, indicating that it is a good strategy in this group of patients.

## Data Availability Statement

The original contributions presented in this study are included in the article/Supplementary Material, further inquiries can be directed to the corresponding author/s.

## Author Contributions

MS-A, CR, and ME contributed to the conception and design of the study. MS-A and CR wrote the first draft of the manuscript. MJS wrote sections of the manuscript. MJS and ME reviewed the manuscript. All authors provided patients data, contributed to manuscript revision, read, and approved the submitted version.

## Conflict of Interest

The authors declare that the research was conducted in the absence of any commercial or financial relationships that could be construed as a potential conflict of interest.

## Publisher’s Note

All claims expressed in this article are solely those of the authors and do not necessarily represent those of their affiliated organizations, or those of the publisher, the editors and the reviewers. Any product that may be evaluated in this article, or claim that may be made by its manufacturer, is not guaranteed or endorsed by the publisher.

## Supplementary Material

Supplementary Figure 1 Patients according to their percentage of crescents.

## References

[1] Pusey CD. Anti-glomerular basement membrane disease. *Kidney Int.* (2003) 64:1535–50. 10.1046/j.1523-1755.2003.00241.x 12969182

[2] Canney M O’Hara PV McEvoy CM Medani S Connaughton DM Abdalla AA Spatial and temporal clustering of anti-glomerular basement membrane disease. *Clin J Am Soc Nephrol.* (2016) 11:1392–9. 10.2215/CJN.13591215 27401523 PMC4974897

[3] McAdoo SP Tanna A Hrušková Z Holm L Weiner M Arulkumaran N Patients double-seropositive for ANCA and anti-GBM antibodies have varied renal survival, frequency of relapse, and outcomes compared to single-seropositive patients. *Kidney Int.* (2017) 92:693–702. 10.1016/j.kint.2017.03.014 28506760 PMC5567410

[4] van Daalen EE Jennette JC McAdoo SP Pusey CD Alba MA Poulton CJ Predicting outcome in patients with anti-GBM glomerulonephritis. *Clin J Am Soc Nephrol.* (2018) 13:63–72. 10.2215/CJN.04290417 29162595 PMC5753308

[5] Charles Jennette J. Rapidly progressive crescentic glomerulonephritis. *Kidney Int.* (2003) 63:1164–77. 10.1046/j.1523-1755.2003.00843.x 12631105

[6] Fischer EG Lager DJ. Anti-glomerular basement membrane glomerulonephritis. *Am J Clin Pathol.* (2006) 125:445–50. 10.1309/NPTP4UKV7JU3ELMQ 16613350

[7] Henderson SR Salama AD. Diagnostic and management challenges in Goodpasture’s (anti-glomerular basement membrane) disease. *Nephrol Dial Transplant.* (2018) 33:196–202. 10.1093/ndt/gfx057 28459999

[8] de Zoysa J Taylor D Thein H Yehia M. Incidence and features of dual anti-GBM-positive and ANCA-positive patients. *Nephrology.* (2011) 16:725–9. 10.1111/j.1440-1797.2011.01484.x 21649794

[9] Jayne DRW Marshall PD Jones SJ Lockwood CM. Autoantibodies to GBM and neutrophil cytoplasm in rapidly progressive glomerulonephritis. *Kidney Int.* (1990) 37:965–70. 10.1038/ki.1990.72 2179617

[10] Verburgh CA Bruijn JA Daha MR van Es LA. Sequential development of anti-GBM nephritis and ANCA-associated pauci-immune glomerulonephritis. *Am J Kidney Dis.* (1999) 34:344–8. 10.1016/S0272-6386(99)70366-5 10430985

[11] Segelmark M Hellmark T Wieslander J. The prognostic significance in Goodpasture’s disease of specificity, titre and affinity of anti-glomerular-basement-membrane antibodies. *Nephron Clin Pract.* (2004) 94:c59–68. 10.1159/000072022 12902632

[12] Levy JB Turner AN Rees AJ Pusey CD. Long-term outcome of anti–glomerular basement membrane antibody disease treated with plasma exchange and immunosuppression. *Annn Intern Med.* (2001) 134:1033. 10.7326/0003-4819-134-11-200106050-00009 11388816

[13] Cui Z Zhao J Jia XY Zhu SN Jin QZ Cheng XY Anti-glomerular basement membrane disease. *Medicine.* (2011) 90:303–11. 10.1097/MD.0b013e31822f6f68 21862934

[14] Touzot M Poisson J Faguer S Ribes D Cohen P Geffray L Rituximab in anti-GBM disease: a retrospective study of 8 patients. *J Autoimmun.* (2015) 60:74–9. 10.1016/j.jaut.2015.04.003 25953709

[15] Kidney Disease Improving Global Outcomes [KDIGO]. Glomerulonephritis work group: KDIGO clinical practice guideline for glomerulonephritis. *Kidney Int Suppl.* (2012) 2:243–51.

[16] Lockwood CM Pearson TA Rees AJ Evans DJ Peters DK Wilson CB. Immunosuppresion and plasma-exchange in the treatment of Goodpasture’s syndrome. *Lancet.* (1976) 307:711–5. 10.1016/S0140-6736(76)93089-0 56532

[17] Johnson JP Moore J Austin HA Balow JE Antonovych TT Wilson CB. Therapy of anti-glomerular basement membrane antibody disease. *Medicine.* (1985) 64:219–27. 10.1097/00005792-198507000-00003 3892220

[18] Kaplan AA. A simple and accurate method for prescribing plasma exchange. *ASAIO Trans.* (1990) 36:M597–9. 2252761

[19] Alchi B Griffiths M Sivalingam M Jayne D Farrington K. Predictors of renal and patient outcomes in anti-GBM disease: clinicopathologic analysis of a two-centre cohort. *Nephrol Dial Transplant.* (2015) 30:814–21. 10.1093/ndt/gfu399 25609740

[20] Merkel F Pullig O Marx M Netzer KO Weber M. Course and prognosis of anti-basement membrane antibody (anti-BM-Ab)-mediated disease: report of 35 cases. *Nephrol Dial Transplant.* (1994) 9:372–6. 8084449

[21] Huart A Josse AG Chauveau D Korach JM Heshmati F Bauvin E Outcomes of patients with Goodpasture syndrome: a nationwide cohort-based study from the French society of hemapheresis. *J Autoimmun.* (2016) 73:24–9. 10.1016/j.jaut.2016.05.015 27267459

[22] Segelmark M Uhlin F Sonesson E. *The Immunoglobulin G Degrading Enzyme Imlifidase for the Treatment of Anti-GBM Disease: The GOOD-IDES 01 Trial.* Washington, DC: American Society of Nephrology (2020).

[23] McAdoo SP Pusey CD. Anti-glomerular basement membrane disease. *Clin J Am Soc Nephrol.* (2017) 12:1162–72. 10.2215/CJN.01380217 28515156 PMC5498345

[24] Bosch X Mirapeix E Font J Borrellas X Rodríguez R López-Soto A Prognostic implication of anti-neutrophil cytoplasmic autoantibodies with myeloperoxidase specificity in anti-glomerular basement membrane disease. *Clin Nephrol.* (1991) 36 107–13. 1657470

[25] Levy JB Hammad T Coulthart A Dougan T Pusey CD. Clinical features and outcome of patients with both ANCA and anti-GBM antibodies. *Kidney Int.* (2004) 66:1535–40. 10.1111/j.1523-1755.2004.00917.x 15458448

[26] Rutgers A Slot M van Paassen P van Breda Vriesman P Heeringa P Tervaert JWC. Coexistence of anti-glomerular basement membrane antibodies and myeloperoxidase-ANCAs in crescentic glomerulonephritis. *Am J Kidney Dis.* (2005) 46:253–62. 10.1053/j.ajkd.2005.05.003 16112043

[27] Weber MF Andrassy K Pullig O Koderisch J Netzer K. Antineutrophil-cytoplasmic antibodies and antiglomerular basement membrane antibodies in Goodpasture’s syndrome and in Wegener’s granulomatosis. *J Am Soc Nephrol.* (1992) 2:1227–34. 10.1681/ASN.V271227 1317224

[28] Lindič J Vizjak A Ferluga D Kovač D Aleš A Kveder R Clinical outcome of patients with coexistent antineutrophil cytoplasmic antibodies and antibodies against glomerular basement membrane. *Ther Apher Dial.* (2009) 13:278–81. 10.1111/j.1744-9987.2009.00724.x 19695059

[29] Tang W McDonald SP Hawley CM Badve SV Boudville NC Brown FG Anti-glomerular basement membrane antibody disease is an uncommon cause of end-stage renal disease. *Kidney Int.* (2013) 83:503–10. 10.1038/ki.2012.375 23254902

[30] Kotanko P Pusey CD Levy JB. Recurrent glomerulonephritis following renal transplantation. *Transplantation.* (1997) 63:1045–52. 10.1097/00007890-199704270-00001 9133463

