# Supplementary figures and images for: Anti-glomerular Basement Membrane Glomerulonephritis: A Study in Real Life
Source: Front Med (Lausanne). 2022 Jul 5;9:889185. doi: 10.3389/fmed.2022.889185 (PMC9295717; doi:10.3389/fmed.2022.889185)

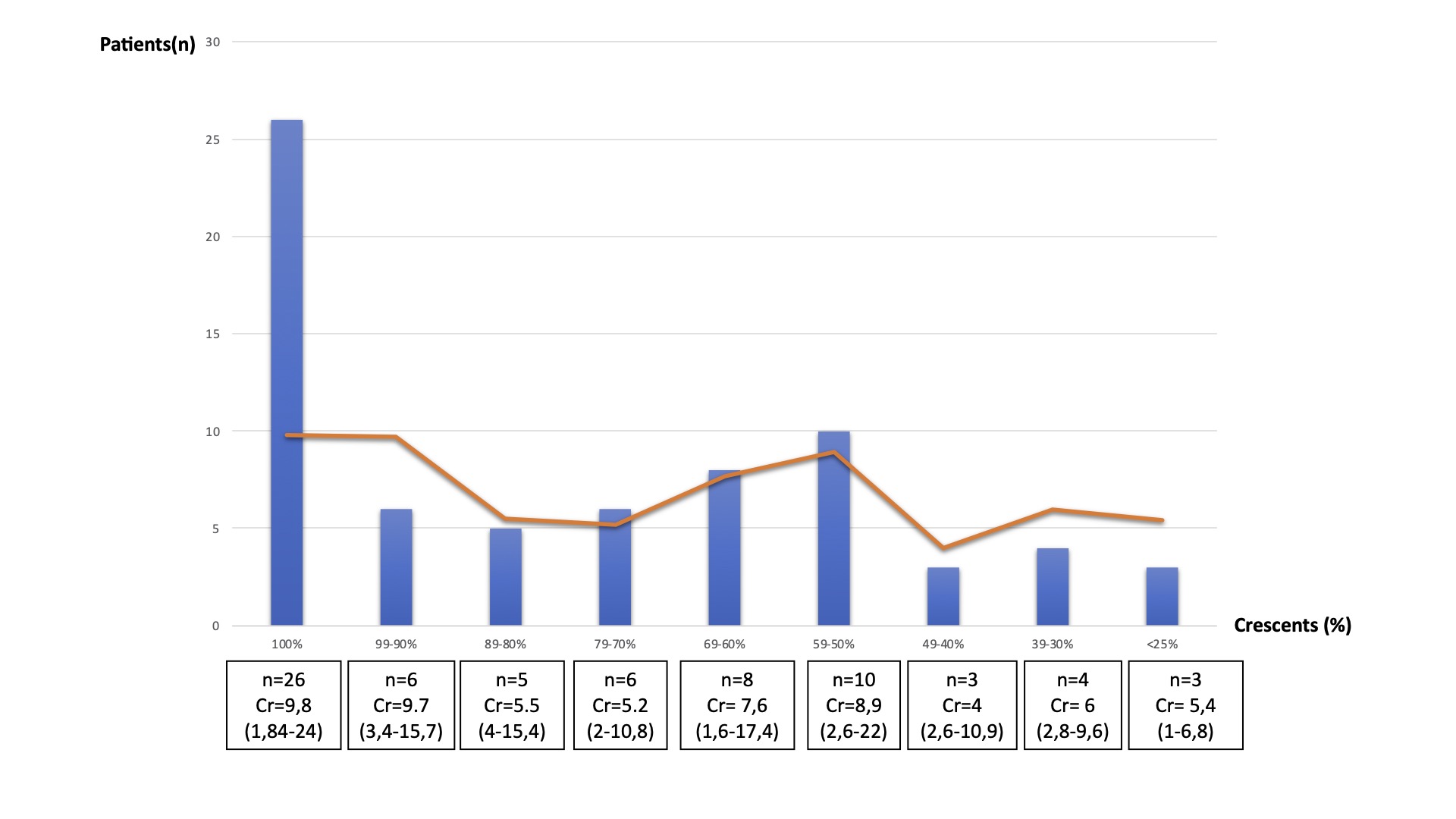

Supplement: Supplementary Figure 1 — Patients according to their percentage of crescents. [file Image_1.JPEG]
